# Serodiagnosis of Primary Infections with Human Parvovirus 4, Finland

**DOI:** 10.3201/eid1701.100750

**Published:** 2011-01

**Authors:** Anne Lahtinen, Pia Kivelä, Lea Hedman, Arun Kumar, Anu Kantele, Maija Lappalainen, Kirsi Liitsola, Matti Ristola, Eric Delwart, Colin Sharp, Peter Simmonds, Maria Söderlund-Venermo, Klaus Hedman

**Affiliations:** Author affiliations: University of Helsinki, Helsinki, Finland (A. Lahtinen, L. Hedman, A. Kumar, A. Kantele, M. Söderlund-Venermo, K. Hedman);; Helsinki University Central Hospital, Helsinki (P. Kivelä, L. Hedman, A. Kantele, M. Lappalainen, M. Ristola, K. Hedman);; National Institute for Health and Welfare, Helsinki (K. Liitsola);; Blood System Research Institute, San Francisco, California, USA (E. Delwart);; University of California, San Francisco (E. Delwart);; University of Edinburgh, Summerhall, UK (C. Sharp, P. Simmonds)

**Keywords:** Parvovirus, viruses, hepatitis C virus, HIV, antibodies, serodiagnosis, primary infection, injection drug users, Finland, dispatch

## Abstract

To determine the prevalence of parvovirus 4 infection and its clinical and sociodemographic correlations in Finland, we used virus-like particle–based serodiagnostic procedures (immunoglobulin [Ig] G, IgM, and IgG avidity) and PCR. We found 2 persons with parvovirus 4 primary infection who had mild or asymptomatic clinical features among hepatitis C virus–infected injection drug users.

A new member of family *Parvoviridae*, human parvovirus 4 (PARV4), was identified in plasma of an injection drug user (IDU) with unexplained fatigue, headaches, fever, night sweats, nausea, and diarrhea (*1*). In PCR studies of blood and postmortem tissues, virus was detected mainly in persons with histories of injection drug use (*2**–**5*). A recent PARV4 immunoglobulin (Ig) G study also showed higher prevalence of antibodies to PARV4 in IDUs and HIV-positive persons who had hemophilia than in HIV-positive men who have sex with men (*6*). The clinical role of this virus is unknown.

We report virus-like particle–based comprehensive serodiagnosis for PARV4 and determine its occurrence in Finland in 3 diverse population groups. In the highest prevalence group, we comparatively analyzed PARV4 IgG–positive and IgG–negative persons for sociodemographic and behavioral background factors and symptoms by using an HIV risk factor database (*7*).

## The Study

Group 1 (low risk) comprised 115 university students (1 serum sample/student). Group 2 (high risk) comprised 78 HIV IgG–positive IDUs from the Helsinki Cohort Study (*8*) (151 plasma samples, 1–7 samples/person). Group 3 (high risk) comprised 200 hepatitis C virus (HCV) IgG–positive patients (1 sample/person). Informed consent was obtained from persons in groups 1 and 2 and from 2 patients with primary infections in group 3. The study was reviewed and approved by the Helsinki University Central Hospital Ethics Committee (#281/13/03/01/09 and #469/2001).

Four genomic regions of open reading frame 2 of PARV4 genotype 1 (AY622943) were cloned for baculovirus expression by using PARV4 PCR–positive plasma (*1*) as initial template. The clone with nt region 3137–5122 (AY622943) and infectivity was constructed with primers PARV4*Eco*RI_3137: 5′-TATGAATTCATGATTGAGCATGGGG-3′ and PARV4EagI_5122: 5′-TACGGCCGTTACAGCAAATGAGAATAA-3′.

**Figure 1:**
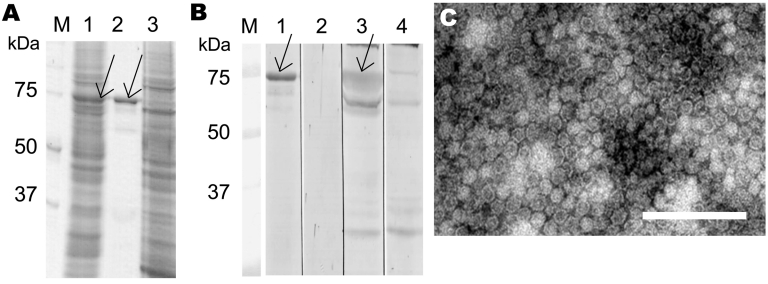
Parvovirus 4 (PARV4) virus-like particle (VLP) expression and immunoreactivity, Finland. A) Sodium dodecyl sulfate–polyacrylamide gel electrophoresis of PARV4-like particles in *Spodoptera frugiperda* armyworm (Sf)9 cells (lane 1), purified VLPs (lane 2), and uninfected Sf9 cells (lane 3). B) Western blotting with PARV4 immunoglobulin (Ig) G–positive serum (lanes 1, 3, and 4) or PARV4 IgG–negative serum (lane 2). Lanes 1 and 2, purified VLPs as antigen; lane 3, Sf9 cells expressing VLPs; lane 4, Sf9 cells expressing glutathione-S-transferase control antigen; lanes M, molecular mass marker. Arrows in panels A and B indicate the PARV4 capsid protein. C) Electron micrograph of purified VLPs. Scale bar = 200 nm.

Protein expression and virus-like particle purification were conducted as for human bocavirus (HBoV) (*9*). Sodium dodecyl sulfate–polyacrylamide gel electrophoresis identified a 73-kDa protein (Figure 1, panel A), which was immunoreactive by Western blotting (*10*) with 5 known PARV4 IgG–positive serum samples (*6*) but not with negative serum samples (Figure 1, panel B). Electron microscopy showed spherical particles ≈25 nm in diameter (Figure 1, panel C) that resembled those seen in vivo (*11*). The capsid protein region is 109 aa longer (N terminally) than that reported by Sharp et al. (*6*); both constructs assembled into capsids.

PARV4 IgG enzyme immunoassay (EIA) was conducted as for HBoV (*9*). Specific results were obtained by subtracting antigen-free background levels. For IgM EIA, a μ-capture format was used (*9*). IgG and IgM cutoff values, obtained from group 1 absorbances (mean + 4 × SD), were 0.141 and 0.205, respectively. IgG-avidity EIA was conducted as for HBoV (method A) (*12*); cutoff values for high and low avidity were 25% and 15%, respectively.

**Figure 2:**
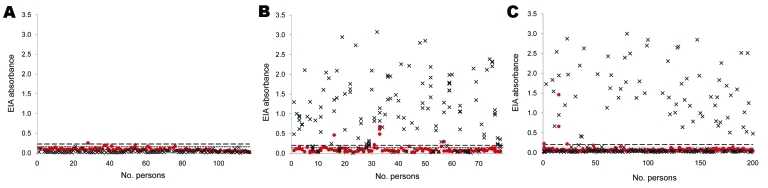
Parvovirus 4 (PARV4) enzyme immunoassay (EIA) results, Finland. Red dots, immunoglobulin (Ig) M; ×, IgG. Upper dashed line indicates IgM cutoff value (0.205), and lower dashed line indicates IgG cutoff value (0.141). A) Group 1: 115 university students (1 serum sample/person); none positive for PARV4 IgG, and 1 positive for PARV4 IgM. B) Group 2: 78 HIV-infected injection drug users (151 serum samples [1–7 samples/person]). Prevalences of PARV4 IgG and IgM were 78.2% (61/78) and 5.1% (4/78), respectively. C) Group 3: 200 hepatitis C virus–infected patients (1 sample/person). Prevalences of PARV4 IgG and IgM were 34.5% (69/200) and 1.5% (3/200), respectively.

None of the 115 students (group 1) were PARV4 IgG positive, and 1 (0.9%) of 115 was weakly IgM positive (Figure 2, panel A). Sixty-one (78.2%) of 78 HIV-infected patients (group 2) were IgG positive, and 4 (5.1%) of 78 were IgM positive (Figure 2, panel B). Sixty-nine (34.5%) of 200 HCV-infected patients (group 3) were IgG positive, and 3 (1.5%) of 200 were IgM positive (Figure 2, panel C). Previous samples were available for 2 of the IgM-positive patients (A and B) in group 3. These samples showed seroconversion for IgG and an increase in IgG (Table 1).

**Table 1:** Virologic findings for PARV4 primary infections in 2 patients, Finland*

Patient	Date of sampling	IgG EIA absorbance	IgM EIA absorbance	PCR	IgG avidity, %
A	2004 Sep 8	0.016	0.051	–	ND
	2006 Mar 14	2.873	0.218	+	17.7
B	2006 Mar 13	0.950	1.461	+	8.9
	2006 Apr 6	1.946	0.661	+	10.1

*PARV4, parvovirus 4; Ig, immunoglobulin; EIA, enzyme inmmunoassay; –, negative; ND, not determined; +, positive.

PARV4 IgG avidity was determined in all persistently (>1 year) IgG-positive persons in group 2 (n = 29). Twenty-eight persons showed high IgG avidity, and 1 showed borderline IgG avidity. All 4 IgM-positive persons had high-avidity IgG, which indicated previous immunity.

In group 3, a second sample from patient A, who showed seroconversion for IgG showed borderline IgG avidity. Patient B showed low IgG avidity in both samples (Table 1).

Groups 2 and 3 were also analyzed for PARV4 DNA by qualitative PCR (*13*) as modified (94°C for 10 min; 45 cycles at 94°C for 20s, 51°C or 56°C for 20s, and 72°C for 20s; and extension at 72°C for 7 min). Amplicons were subjected to electrophoresis and sequenced. In group 2, all 151 serum samples were PCR negative. In group 3, two patients (A and B) were PCR positive (Table 1).

PARV4 IgG–positive and IgG–negative IDUs (group 2) were compared for demographic and clinical characteristics. PARV4 IgG–positive persons reported more injection of drugs, persistent (>10 y) injection, and lending of injection equipment (Table 2). They also had a more frequent history of imprisonment and unemployment and were less educated. No differences were seen between PARV4 IgG–positive and IgG–negative persons with any symptoms (fever, tiredness, nocturnal sweating, cough, diarrhea, shortness of breath, swallowing complaints, muscle weakness, dizziness, skin abscesses or herpetic lesions, loss of eyesight, or headache) during 6 months before being interviewed.

**Table 2:** Characteristics of PARV4 IgG–positive and IgG–negative HIV-infected injection drug users, Finland*

Characteristic	IgG positive, n = 61	IgG negative, n = 17	p value†
Age, y, median (range)	35 (17–61)	31 (21–55)	0.069
Male sex	44/61 (72)	12/17 (71)	1.000
Main drug was amphetamine‡	38/61 (62)	10/17 (59)	0.786
Duration of injection, y, median (range)	10 (0–36)	7 (0–30)	0.259
Duration of injection >10 y	45/59 (76)	7/17 (44)	**0.029**
History of imprisonment	49/61 (80)	9/17 (53)	**0.031**
Education <9 y	52/59 (88)	10/17 (59)	**0.011**
HCV antibody positive	59/60 (98)	14/15 (93)	0.362
HBsAg positive	3/61 (5)	3/17 (18)	0.114
HBc IgG positive	46/60 (77)	9/17 (56)	0.124
Used antiretroviral therapy§	29/61 (48)	4/17 (24)	0.099
CD4 cell count/µL, median§	303	323	0.168
Present situation			
Unstable living conditions (no permanent address)	35/59 (60)	5/15 (33)	0.088
Employed‡	0/53 (0)	3/16 (19)	**0.009**
Risk behavior			
Loaned needles or syringes	49/59 (83)	9/16 (56)	**0.040**
Borrowed needles or syringes	56/58 (97)	15/16 (94)	0.524
Had sexually transmitted diseases	37/57 (65)	7/17 (41)	0.097
Had commercial sex	18/61 (30)	8/15 (53)	0.127
Risk behavior past 6 mo			
Used drugs	48/61 (79)	8/17 (47)	**0.016**
Used injection drugs	46/61 (75)	8/17 (47)	**0.037**
Used condoms inconsistently	25/61 (41)	7/16 (44)	1.000
Had >2 sex partners	16/61 (26)	7/17 (41)	0.244

*Values are no. positive/no. tested (%) unless otherwise indicated. PARV4, parvovirus 4; Ig, immunoglobulin; HCV, hepatitis C virus; HBsAg, hepatitis B surface antigen; HBc, hepatitis B core antigen; †By Fisher exact test or Mann-Whitney U test. Values in **boldface** are significant. ‡At the time of interview. §Closest to the sampling for PARV4 tests.

## Conclusions

We developed IgG-, IgM-, and IgG-avidity–based PARV4 serodiagnostic procedures; studied high-prevalence cohorts by PCR; and analyzed HIV-infected IDUs for demographic and clinical correlations with PARV4 IgG positivity. Among healthy university students, none had PARV4 IgG, which is consistent with low baseline IgG prevalences of 0% and 2.8% for another EIA (*6*). The PARV4 IgG seroprevalence of 78% among HIV-infected IDUs represents a high incidence of PARV4, which reflects the lengthy history of drug use among socially marginalized IDUs during an HIV outbreak in Finland (*7*).

Two HCV-infected patients had PARV4 primary infections, as shown by increasing IgG levels, detectable IgM, low or borderline IgG avidity, and viral DNA in serum. These 4 findings are presented as diagnostic criteria for PARV4 primary infection. As estimated by known kinetics of B19 virus diagnostics (*14*), these 2 PARV4 infections probably occurred in 2005. During that time, neither patient had contacted local healthcare providers. Conversely, these 2 patients used intravenous drugs daily, and might not have sought medical care unless they were severely ill.

Because PARV4 IgG seroprevalence in group 1 was 0% in this study, in contrast to prevalences of 60% for B19 (*12*) and 96% for HBoV (*9*) in the same students, serologic cross-reactivity between PARV4 and the other human parvoviruses appears highly unlikely. Amino acid sequence similarity is <30% between B19 and PARV4 and ≈40% between HBoV and PARV4.

PCR-negative results for group 2, including 4 patients who were IgM positive, are evidence against viremic primary, chronic, and recurrent PARV4 infections. However, because of the relatively low sensitivity of this PCR, the data do not rule out low levels of viral DNA in blood.

Analysis of HIV-infected IDUs supports the view that in northern Europe PARV4 is primarily a blood-borne virus. No differences were seen for factors related to sexual activity. However, our sample size was too small to make this conclusion. In a recent PCR study, PARV4 genotype 3 was commonly found among infants in West Africa, and there was no evidence of parenteral exposure (*15*).

Using comprehensive serodiagnosis, we showed that PARV4 is ubiquitously present in IDUs in Finland and detected primary infections in 2 patients who had a full spectrum of diagnostic findings. Neither of these had sought medical help, which suggested that their primary infections may have been clinically mild or asymptomatic.
